# Safety and Efficacy of the COVID-19 Vaccine in Kidney Transplant Recipients

**DOI:** 10.7759/cureus.24753

**Published:** 2022-05-05

**Authors:** Abdulrahman Altheaby, Duha Alloqmani, Rawaby AlShammari, Albatoul Alsuhaibani, Anadel Hakeem, Syed Alam, Shroug Alharbi, Mohammed Al Zunitan, Mohammad Bosaeed, Naif K Alharbi

**Affiliations:** 1 Department of Hepatobiliary Sciences and Organ Transplant Center, King Abdulaziz Medical City, Riyadh, SAU; 2 Department of Medicine, King Abdulaziz Medical City, Riyadh, SAU; 3 Division of Infectious Diseases, Department of Internal Medicine, King Abdulaziz Medical City/National Guards Health Affairs, Riyadh, SAU; 4 Infectious Diseases Research, King Abdullah International Medical Research Center, Riyadh, SAU

**Keywords:** coronavirus disease 2019 (covid-19), igg antibodies, humoral immunogenicity, covid-19 vaccination, kidney transplant recipients

## Abstract

Background: Kidney transplant recipients appear to be at high risk for critical coronavirus disease 2019 (COVID-19) illness. They are considered a priority for COVID-19 vaccination. Only a few studies report on the safety and efficacy of the COVID-19 vaccine in these patients.

Methods: In this prospective observational study, we measured anti-severe acute respiratory syndrome coronavirus 2 (anti-SARS-CoV-2) spike-specific IgG post first and second COVID-19 mRNA vaccines in 113 kidney transplant recipients and compared them to 62 healthy volunteers.

Result: After the first COVID-19 vaccine, SARS-CoV-2-specific antibodies were undetectable in 38.9% of kidney transplant recipients, and after the second, it remained undetectable in 12.4%. SARS-CoV-2-specific antibodies were significantly lower in kidney transplant recipients. The average antibody titer after the first vaccine was 1243.6±4137.5 in kidney transplant recipients compared to 20012.2±44436.4 in the controls after the first dose (P=0.002), and 7965.5±12431.3 versus 82891.3±67418.7, respectively, after the second dose (P <0.001). The immune response to the COVID-19 vaccine seemed to be influenced by mycophenolate dose in kidney transplant recipients and pre-vaccination infection.

Conclusion: Kidney transplant recipients are prone to have attenuated antibody responses (anti-spike IgGs) to mRNA COVID-19 vaccines. Patients on mycophenolate mofetil (2 gm daily) had significantly lower SARS-CoV-2 spike-specific IgG levels as compared to patients on no or reduced dose of mycophenolate. Hence, kidney transplant recipients need to continue all infection control precautionary measures against COVID-19 infection and should be considered a priority for a third COVID-19 vaccine.

## Introduction

Coronavirus disease 2019 (COVID-19) has infected more than 270 million individuals globally since it was declared a pandemic by the World Health Organization on March 11, 2020 [1]. The disease is caused by severe acute respiratory syndrome coronavirus 2 (SARS-CoV-2), which is an enveloped single positive-stranded RNA virus with helical capsids [2]. Coronaviruses are known to infect a wide range of hosts including mammals and birds. SARS-CoV-2, SARS-CoV-1 identified in 2003, and the Middle East respiratory syndrome coronavirus (MERS-CoV) that emerged in 2012 all belong to the same betacoronavirus genus [2].

The clinical manifestation of COVID-19 is highly variable. It ranges from asymptomatic infection to acute respiratory distress syndrome (ARDS) and multi-organ dysfunction, especially in high-risk populations [3]. Kidney transplant recipients are considered at high risk for critical COVID-19 illness due to chronic use of immunosuppressive medications [4]. Mortality rates of COVID-19 infection in kidney transplant recipients have varied in studies, ranging from 7.1% to as high as 53% [5]. In Saudi Arabia, 55% of kidney transplant recipients with COVID-19 infection required hospitalization, with a 10.8% mortality rate [6]. In order to protect this high-risk population, solid organ transplant recipients have been considered a priority for vaccination programs by the health authorities of many countries [7].

The use of micro-ribonucleic acid (mRNA) vaccines in kidney transplant recipients are generally safe [8]. It acts by delivering modified messenger RNA into the host cells to allow expression of the SARS-CoV-2 spike (S) antigen. This antigen will stimulate both humoral and cellular immune responses to the S antigen [9]. It is not yet entirely clear whether protection against COVID-19 after mRNA vaccination depends mainly on the cellular or humoral immune response. However, recent reports have clearly demonstrated the importance of developing neutralizing antibodies that bind to the spike protein of SARS-CoV-2 to eliminate viruses and prevent their entry into host cells [10]. The measurement of anti-spike IgGs can be performed more easily by a widely available commercial serological test than by measuring neutralizing antibodies. Anti-spike IgGs correlate well with the presence of neutralizing antibodies and vaccine efficacy against primary symptomatic COVID-19 [11].

Few recent studies have reported the humoral and cell-mediated immune responses to mRNA-based COVID-19 vaccines and their safety profile in kidney transplant recipients. These reports suggested attenuated antibody responses (anti-spike IgGs) to COVID-19 vaccines in kidney transplant recipients. Detectable anti-spike IgGs in kidney transplant recipients range from 0% to 17% following one COVID-19 vaccine dose, and 3% to 59% after two COVID-19 vaccine doses [12]. This demonstrates that two COVID-19 vaccines are insufficient to protect kidney transplant recipients as they remain at a risk for severe cases of COVID-19 illness despite two vaccine doses [12]. Consequently, kidney transplant recipients are prioritized for an extra boosting dose of the COVID-19 vaccine [13].

In this prospective study, we report the safety, immunogenicity, and efficacy of SARS-CoV-2 vaccination in kidney transplant recipients and compare them to a vaccinated healthy control group.

## Materials and methods

This is a prospective observational study, enrolling all kidney transplant recipients (age > 18 years old) in King Abdulaziz Medical City (KAMC), Riyadh, Saudi Arabia, who received at least one dose of either BNT162b2 (Pfizer/BioNTech, Pfizer Inc., New York, United States /BioNTech SE, Mainz, Germany), AZD1222 (Oxford/AstraZeneca, University of Oxford, Oxford, United Kingdom/AstraZeneca plc, Cambridge, United Kingdom), or JNJ-78436735 (Johnson & Johnson, New Brunswick, New Jersey, United States) vaccine between January and July, 2021. The study was reviewed and approved by the Institutional Ethics Review Board of King Abdullah International Medical Research Center (KAIMRC) with Memo Ref. No. IRBC/0974/21. All enrolled participants (n=113) signed a written informed consent form. Clinical data were collected from electronic medical records. In addition, separate questionnaires were used to survey patients for any adverse reactions after vaccination, and any history of SARS-CoV-2 infection pre- or post-vaccination. Two blood samples were taken for each consenting patient to test their humoral response to the SARS-CoV-2 vaccine. Samples were collected two to four weeks after the first dose and four to eight weeks after the second dose. Blood testing for kidney function and drug levels and urine analysis were documented as well. A group of healthy individuals (n=62) who received COVID-19 vaccines were sampled and included as a control in the vaccine humoral immunogenicity testing. Patients younger than 18 years old, patients with recent history (within six months) of cellular or humoral acute rejection, patients with cancer requiring radiotherapy or chemotherapy during the last six months, and patients who are not on regular follow-up were excluded.

Humoral immunogenicity

Enzyme-linked immunosorbent assay (ELISA) was conducted in-house to detect SARS-CoV-2 IgG for serum samples according to the previously published protocols [14]. Recombinant S1 subunit of the SARS-CoV-2 spike protein (Sino Biological, Beijing, China) was used to coat Nunc MaxiSorp 96-well ELISA microplates (Thermo Fisher, Waltham, Massachusetts, United States) at a concentration of 1 ug/mL and incubated overnight at 4^◦^C. The following day, the plates were washed with washing buffer (phosphate-buffered saline (PBS) with 0.5% Tween20 (PBS-T)) using an automated Microplate Washer (Molecular Devices, San Jose, California, United States) and blocked with blocking buffer (washing buffer containing 10% skimmed milk) at 37^◦^C for one hour. Plates were then washed, serum samples diluted in a three-fold serial dilution starting from a 1:100, and PBS-T were added into duplicate wells and incubated for two hours. Following the wash, plates were incubated with alkaline phosphatase labeled “goat anti-human IgG secondary antibody” (Thermo Fisher, Waltham, Massachusetts, United States) at 37^◦^C for one hour. After the wash, the plates were incubated with p-Nitrophenyl Phosphate, Disodium Salt (PNPP) substrate at 37^◦^C for 30 minutes. The optical density (OD) was measured at 405 nm using Microplate Reader (Molecular Devices, San Jose, CA). The endpoint titer for each tested serum was determined as the reciprocal value of the serum dilution with the OD value converging with the cut-off. The cut-off was determined as the average OD of the negative control serum + 3 serial dilution [15].

Statistical analysis

We used IBM SPSS Statistics for Windows, Version 24.0 (Released 2016; IBM Corp., Armonk, New York, United States) for the analysis. Continuous variables are denoted as mean ± SD if normally distributed, or median (interquartile range) for non-normal distribution. The Shapiro-Wilk test was used to examine the normality of continuous variables and guide the selection of a parametric or nonparametric test for the comparison of variables. The variables were compared using Welch’s t-test, Student t-test, and Mann-Whitney-U test. "General" linear model (GLM) repeated measures, ANOVA was used to assess within-subject longitudinal effects for changes in creatinine, WBC, and calcineurin inhibitor (CNI). Categorical variables are presented as frequencies and percentages and were compared using the Chi-squared test, Fisher's exact test, or McNemar's test as appropriate. All reported P-values are two-sided and P-values < 0.05 were considered to indicate a statistical significance.

## Results

A total of 113 kidney transplant recipients and 62 healthy volunteers were enrolled in this prospective observational study. The average age in the transplant group was 45.1±16.2 years and that in the control group was 45.7±13.9 years (P= 0.783). The male gender was more prevalent in the transplant group while the majority of controls were females (P=0.006). Comorbidities such as diabetes, hypertension, and coronary artery disease were significantly higher in the transplant group as compared to the control. In kidney transplant recipients, 94 (83.2%) patients received a kidney from living donors while 19 (16.8%) patients received a kidney from deceased donors. A total of 67 (59.3%) kidney transplant recipients had anti-thymocyte globulin (ATG) as induction therapy, and basiliximab was the induction agent in 46 (40.7%). The majority of transplant recipients used prednisolone 5 mg, tacrolimus, and mycophenolate as a maintenance regimen. There are 11 (9.7%) kidney transplant recipients not on mycophenolate, and more than 50% of transplant recipients received a reduced dose of mycophenolate (Table 1).

**Table 1:** Baseline characteristics of the study participants DM: diabetes mellitus; HTN: hypertension; CAD: coronary artery disease; ATG: antithymocyte globulin *  Mean ± SD; ^a^ Student t-test; ^b^ Fisher's Exact test; ^c^ Chi-squared test

	Total (n=175)	Transplant patients (N=113)	Control (n=62)	P-value
Age	45.3±15.3^*^	45.1±16.2	45.7±13.9	0.783^ a^
Gender				
Male	101 (57.7%)	74 (65.5%)	27 (43.5%)	0.006^ b^
Female	74 (42.3%)	39 (34.5%)	35 (56.5%)	
BMI	27.8±7.7	28±5.2	27.5±10.9	0.686^ b^
DM	61 (34.9%)	46 (40.7%)	15 (24.2%)	0.032^ b^
HTN	92 (52.6%)	67 (59.3%)	25 (40.3%)	0.018^ b^
Blood group				
A	43 (24.6%)	24 (21.2%)	19 (30.6%)	
AB	8 (4.6%)	6 (5.3%)	2 (3.2%)	0.296^ c^
B	39 (22.3%)	23 (20.4%)	16 (25.8%)	
O	85 (48.6%)	60 (53.1%)	25 (40.3%)	
Smoker	12 (6.9%)	5 (4.4%)	7 (11.3%)	0.117^ b^
CAD	23 (13.1%)	20 (17.7%)	3 (4.8%)	0.018^ b^
Donor				
Deceased		19 (16.8%)		
Living		94 (83.2%)		
Induction				
ATG		67 (59.3%)		
Basiliximab		46 (40.7%)		
Maintenance				
Predo		112 (99.1%)		
Tacrolimus		110 (97.3%)		
Mycophenolate				
1000		48 (42.5%)		
1500		14 (12.4%)		
2000		40 (35.4%)		
No		11 (9.7%)		
Azathioprine		6 (5.3%)		
Tacrolimus		19 (16.8%)		

The BNT162b2 was the most utilized vaccine in 140 (80%) participants, followed by the AZD1222 vaccine in 31 (17.7%) participants. Three participants received AZD1222 as a first dose and BNT162b2 as a second dose. Only one participant received the JNJ-78436735 vaccine. There were no differences in the vaccination distribution between transplant and control groups. In kidney transplant recipients, the average time between the doses was 78.6±61.6 days, and the average time from transplant to first dose was 915.1±960.4 days. Similar adverse event profiles were observed in the two vaccinated groups, except for a significantly higher diarrhea incidence (P= 0.003) in the control group (Table 2).

**Table 2:** Type of vaccine and adverse events Tx: transplant *  Mean ± SD; ^b^ Fisher's Exact test; ^c^ Chi-squared test

	Total (n=175)	Transplant patients (n=113)	Control (n=62)	P-value
Vaccine Type				
AstraZeneca	31 (17.7%)	16 (14.2%)	15 (24.2%)	0.197^ c^
Pfizer	140 (80%)	93 (82.3%)	47 (75.8%)	
AstraZeneca, Pfizer	3 (1.7%)	3 (2.7%)	0 (0%)	
Johnson & Johnson	1 (0.6%)	1 (0.9%)	0 (0%)	
Time between doses (days)		78.6±61.6		
Time from Tx to first dose (days)		915.1±960.4		
Side effects				
Fever	44 (25.1%)	27 (23.9%)	17 (27.4%)	0.716^ b^
Headache	18 (10.3%)	11 (9.7%)	7 (11.3%)	0.797^ b^
Myalgia	39 (22.3%)	22 (19.5%)	17 (27.4%)	0.257^ b^
Abdominal pain	0 (0%)	0 (0%)	0 (0%)	
Diarrhea	8 (4.6%)	1 (0.9%)	7 (11.3%)	0.003^ b^

A longitudinal follow-up of serum creatinine (µmol/L), WBC, urine protein, and urine RBC of kidney transplant recipients after vaccination showed no significant difference as compared to pre-vaccination values. Only tacrolimus trough level dropped with a statistical significance, but it remained within the therapeutic range (Table 3). 

**Table 3:** Laboratory testing for kidney transplant patient * Mean ± SD; ^a^ Paired t-test; ^b^ McNemar's test

	Baseline	After first vaccine	After secondvaccine	Difference	P-value
Creatinine	94.7±26.2^* ^	107.7±134.3	105.2±92.7	10.49 (-5.72 to 26.69)	0.202^ a^
Tacrolimus level	7.1±1.4	7±1.8	6.9±1.5	-0.26 (-0.51 to -0.01)	0.040^ a^
WBC	6.8±2.1	6.8±2.1	7.1±2.2	0.29 (0 to 0.58)	0.053^ a^
Urine Protein	10 (8.8%)	15 (13.3%)	10 (8.8%)	0% (-7.8% to 7.8%)	1^ b^
Urine RBC	11 (9.7%)	14 (12.4%)	12 (10.6%)	0.9% (-7.3% to 9.1)	1^ b^

We documented the COVID-19 infection rate before and after vaccination. The infection rate was significantly higher in kidney transplant recipients before vaccination (P < 0.028). Post-vaccination, there were only four breakthrough COVID-19 cases in kidney transplant recipients. The infection occurred after 55.3±12.3 days from the second dose, and only one patient required ICU care. There was no reported infection in the control group (Table 4).

**Table 4:** COVID-19 cases before and after COVID-19 vaccination among the study participants COVID-19: coronavirus disease 2019 Time from 1st dose to infection: 116.5±27.2* days Time from 2nd dose to infection: 55.3±12.3  days * Mean ± SD; ^a^ Fisher's Exact test

	Total (n=175)	Transplant patients (n=113)	Control (n=62)	P-value
Infected before vaccine	9 (5.1%)	9 (8%)	0 (0%)	<0.028^ a^
presence of symptoms	8 (88.9%)	8 (88.9%)	0 (0%)	
Admission	6 (66.7%)	6 (66.7%)	0 (0%)	
ICU	2 (22.2%)	2 (22.2%)	0 (0%)	
Infected after first dose	0 (0%)	0 (0%)	0 (0%)	
Infected after second dose	4 (2.3%)	4 (3.5%)	0 (0%)	0.299^ a^
Presence of symptoms	4 (100%)	4 (100%)	0 (0%)	
Admission	2 (50%)	2 (50%)	0 (0%)	
ICU	1 (25%)	1 (25%)	0 (0%)	

**Figure 1:**
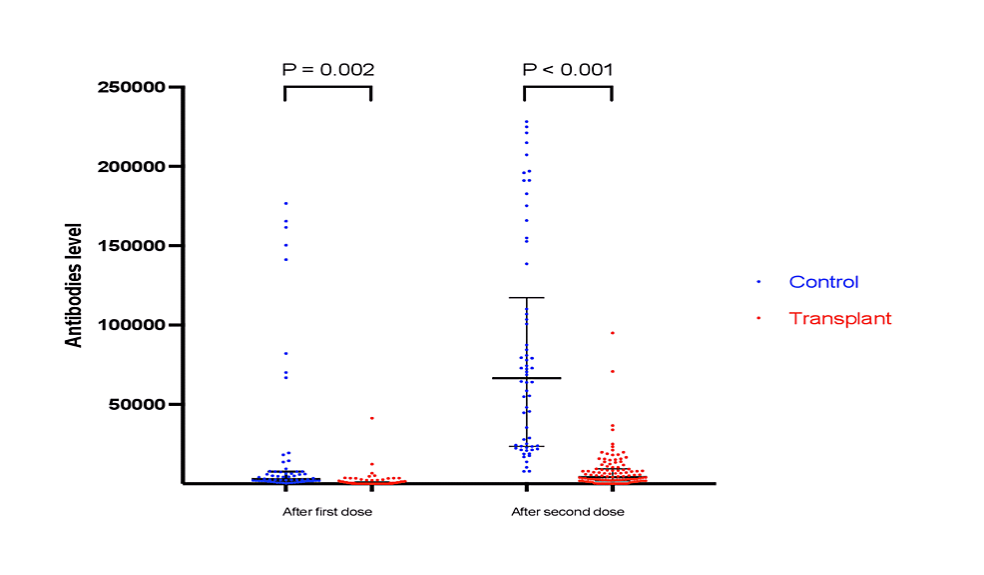
Anti-SARS-CoV-2 spike antibodies in the study participants after each dose of COVID-19 vaccines: Median and interquartile range of antibody titers are shown for the vaccinated healthy control group (blue) and kidney transplant recipients (red) SARS-CoV-2: severe acute respiratory syndrome coronavirus 2; COVID-19: coronavirus disease 2019

Average antibody titer was significantly lower in the transplant group 1243.6±4137.5 as compared to 20012.2±44436.4 in the controls after the first dose (P=0.002); and 7965.5±12431.3 versus 82891.3±67418.7, respectively, after the second dose (P <0.001). Additionally, the SARS-CoV-2 IgG seroconversion rate in kidney transplant recipients was significantly lower. After the first vaccine dose, 38.9% of kidney transplant recipients had no detectable antibodies and it remained undetectable in 12.4% after the second dose, whereas all control groups showed positive seroconversion after the first dose (Table 5, Figure 1).

**Table 5:** SARS-CoV-2 spike-specific antibody titer post-COVID-19 vaccination SARS-CoV-2: severe acute respiratory syndrome coronavirus 2; COVID-19: coronavirus disease 2019 *  Mean ± SD; ^a^ Student t-test; ^b^ Fisher's Exact test

	Total (n=175)	Transplant patients (n=113)	Control (n=62)	P-value
Titer				
Post first dose	7893±28005.5*	1243.6±4137.5	20012.2±44436.4	0.002^ a^
Post second dose	34510.7±54631.3	7965.5±12431.3	82891.3±67418.7	<0.001
Seroconversion (post first dose)				
Positive	131 (74.9%)	69 (61.1%)	62 (100%)	<0.001^ b^
Negative	44 (25.1%)	44 (38.9%)	0 (0%)	
Seroconversion (post second dose)				
Positive	161 (92%)	99 (87.6%)	62 (100%)	0.002 ^b^
Negative	14 (8%)	14 (12.4%)	0 (0%)	

**Figure 2:**
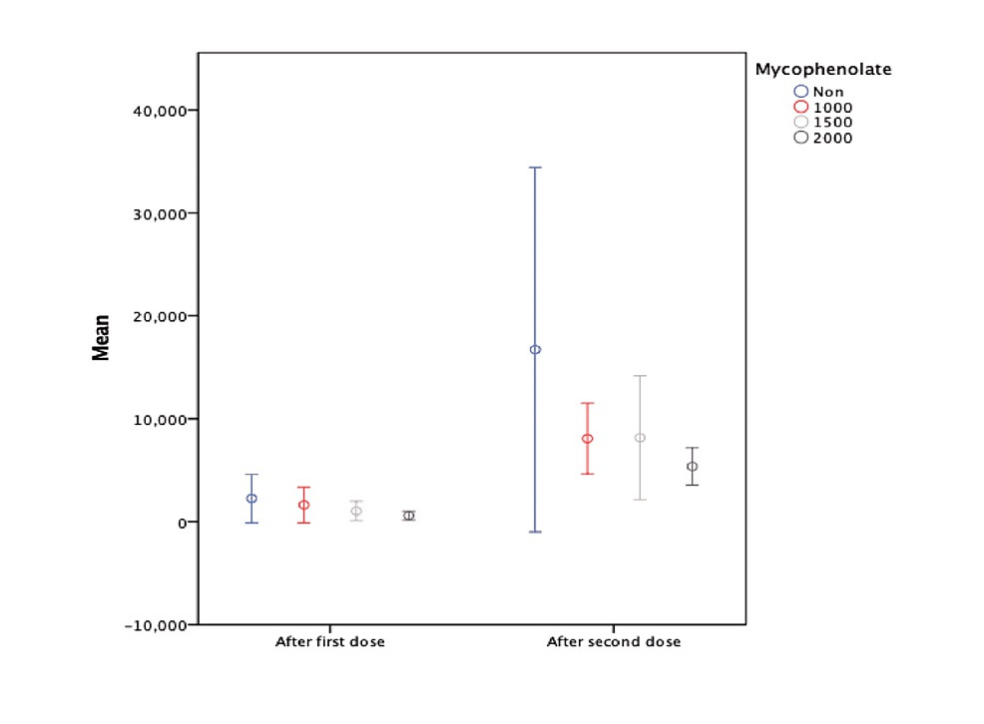
Anti-SARS-CoV-2 spike antibodies in the study participant after each dose of COVID-19 vaccines according to different dosage of mycophenolate. SARS-CoV-2: severe acute respiratory syndrome coronavirus 2; COVID-19: coronavirus disease 2019

An analysis of univariate linear regression has been conducted for all variables in the study including age, gender, BMI, diabetes, HTN, time from transplant to the second vaccine dose, donation type, induction treatment, and maintenance therapy. Only mycophenolate dose and pre-vaccination infection showed significant effects on antibody titer. Consequently, multiple linear regression analysis was performed on mycophenolate dose and pre-vaccination infection which confirm their significant effects on antibody titer (Table 6). Mycophenolate (β = -0.226, P=0.006) and pre-vaccination infection (β = 0.468, p<0.001) were significantly associated with lower and higher post-vaccination antibody titers respectively. Patients who were on mycophenolate 2000 mg daily had lower antibody titers after the COVID-19 vaccination (16719±26369.7) as compared to those who were not taking mycophenolate (5364.5±5675.5) (P=0.044) (Figure 2).

**Table 6:** Independent study variables associated with antibody titer after the second dose, in transplant patients only Basl: basiliximab; ATG: anti-thymocyte globulin; Tx: transplant; DM: diabetes mellitus; HTN: hypertension; CAD: coronary artery disease; SE: standard error; B: unstandardized coefficients; Beta: standardized regression coefficients

	Univariate				Multivariate			
	Unstandardized		Standardized		Unstandardized		Standardized	
	B	SE	Beta	P-value	B	SE	Beta	P-value
Age	-21.383	73.029	-0.028	0.77				
Female vs male	-1343.76	2467.613	-0.052	0.587				
BMI	316.748	226.651	0.131	0.165				
Basi vs. ATG	2771.681	2376.532	0.11	0.246				
Duration from Tx	3897.897	2534.386	0.144	0.127				
Living vs. Deceased	3595.781	3122.373	0.109	0.252				
DM	-879.59	2389.59	-0.035	0.714				
HTN	1041.574	2389.003	0.041	0.664				
CAD	-2694.259	3067.213	-0.083	0.382				
Smoker	-3474.906	5702.727	-0.058	0.544				
Previous infection	21549.325	3826.407	0.471	<0.001	21408.258	3715.901	0.468	<0.001
Prednisolone	2759.973	12540.097	0.021	0.826				
Mycophenolate	-9698.458	3854.456	-0.232	0.013	-9431.332	3393.952	-0.226	0.006
Azathioprine	736.209	5238.398	0.013	0.888				
Tacrolimus	-5243.664	7290.174	-0.068	0.473				

## Discussion

In this prospective observational study, we report an impaired immune response to BNT162b2, AZD1222, or JNJ-78436735 COVID-19 vaccines in kidney transplant recipients compared to healthy vaccinated controls. We analyzed the humoral immune response to COVID-19 vaccination by measuring anti-SARS-CoV-2 spike-specific IgG levels post first and second vaccine doses in kidney transplant recipients and healthy participants.

In general, kidney transplant recipients are known to have impaired humoral and cellular immunity due to chronic use of immunosuppressive medications [16]. They are expected to have a weak immune response to different vaccines, such as hepatitis B or influenza virus [17]. In our study, the vaccine humoral immunogenicity in kidney transplant recipients was measured two to four weeks after the first dose, and four to eight weeks after the second dose, a time point when full antibody response is expected [18]. We demonstrate that after the first vaccine dose, 38.9% of kidney transplant recipients failed to develop spike-specific IgG antibodies above the predetermined cut-off value. Importantly, the antibody titers remained below the cut-off in 12.4% of transplant recipients after the second vaccine dose, indicating a need for a more effective vaccination regimen for this population. By contrast, humoral responses were observed in 100% of controls after the first and second doses. The average antibody titer was significantly lower in transplant recipients as compared to controls after both first and second doses. Our data are in line with other studies that reported poor antibody responses to COVID-19 vaccines in kidney transplant recipients. AlShaqaq et al. reported that only 23.6% of kidney transplant recipients developed anti-spike antibodies after one vaccine dose, and 35.8% showed a positive response following the second dose [19]. Boyarsky et al. reported that 82.6% of transplant recipients (n=436) did not mount significant anti-spike antibody titers after the first dose of mRNA vaccine [20]. Bertrand et al. also reported that only 17.8% of kidney transplant recipients developed anti-spike SARS-CoV-2 antibodies after the second dose [12]. Several other studies reported significantly lower IgG titers in response to the COVID-19 vaccine in kidney transplant recipients [21,22].

The humoral response to the COVID-19 vaccine in kidney transplant recipients is influenced by the intensity of immunosuppressive treatment [23]. In our cohort, most transplant recipients were on prednisolone 5 mg daily and tacrolimus. The dose of tacrolimus was adjusted to keep the trough level between 5-8 ng/mL. Nevertheless, there was inconsistency in using mycophenolate mofetil among transplant recipients. Our multivariate analysis showed that patients on mycophenolate 2 gm daily had significantly lower SARS-CoV-2 spike-specific IgG levels which agrees with other studies that confirmed the negative impacts of intense immunosuppression on IgG titer [24]. The immunosuppression regimen for kidney transplant recipients usually includes drugs that block T cells activation (calcineurin-inhibitor), and drugs that block the proliferation of adaptive immune effectors (mycophenolate mofetil). Studies that assessed immunosuppressant medications' impact on the humoral response to vaccination showed negative effects, especially with mycophenolate mofetil. That has been linked to its role in blocking the proliferation of follicular helper CD4+ T (TFH cells) which is instrumental for the differentiation of spike-specific B cells into antibody-producing plasma cells [25,26,27].

Furthermore, our multivariate analysis showed significantly higher levels of SARS-CoV-2 spike-specific IgG titer in transplant recipients who had COVID-19 prior to vaccination. Similar findings from previous reports have shown that previous COVID-19 infection is an independent predictor of seroconversion post-vaccination [19].

In our cohort, only four kidney transplant recipients had COVID-19 post-second vaccine dose, and only one of them required ICU admission. Our sample size is small to give a conclusion about the protective efficacy of vaccination in kidney transplant recipients, however, recent studies have reported serious COVID-19 infections after two doses of the vaccine [28].

Post-vaccination reactions were assessed in the two groups for BNT162b2, AZD1222, and JNJ-78436735 COVID-19 vaccines. We had only one patient on the JNJ-78436735 vaccine, which is not enough to measure its potential adverse events. In general, vaccines were well tolerated with no major adverse reactions in all participants. The safety profile of the COVID-19 vaccine is consistent with the results of previous studies [29]. However, significantly higher diarrhea incidence occurred in the healthy control group, for which further studies are needed to investigate the possible causes. Changes in graft function post-vaccination were monitored by evaluating the level of creatinine, urine analysis, and tacrolimus trough. There were no significant changes in creatinine level or urine analysis parameters, but tacrolimus trough level has been decreased post vaccination but is still within the therapeutic range. It is unlikely that these changes are related to COVID-19 vaccines, but the effect of COVID-19 vaccines on tacrolimus levels has not been examined, as per our knowledge. 

The current study adds to the existing evidence on COVID-19 vaccine outcomes in kidney transplant recipients, and it has several strengths. It is a prospective study that involved a control group of comparable age and gender to the kidney transplant recipients group. Samples from all patients were analyzed in the same laboratory using an identical protocol for quantification of IgG levels, and a similar protocol for all times. On the other hand, this study also has some limitations. First is the relatively small sample size. Second, kidney transplant recipients have more comorbidities besides kidney transplantation such as diabetes, hypertension, and coronary artery disease. Finally, it was not technically possible to investigate cellular immune responses, which may play an essential role in protection.

## Conclusions

Kidney transplant recipients are prone to have attenuated antibody response (anti-spike IgGs) to mRNA COVID-19 vaccines. Patients on mycophenolate mofetil 2 gm daily had significantly lower SARS-CoV-2 spike-specific IgG levels as compared to the patient on no or reduced dose of mycophenolate. Kidney transplant recipients need to continue all infection control precautionary measures against COVID-19 infection, and should be considered as a priority for a third dose of COVID-19 vaccine.
